# Instability of default mode network connectivity in major depression: a two-sample confirmation study

**DOI:** 10.1038/tp.2017.40

**Published:** 2017-04-25

**Authors:** T Wise, L Marwood, A M Perkins, A Herane-Vives, R Joules, D J Lythgoe, W-M Luh, S C R Williams, A H Young, A J Cleare, D Arnone

**Affiliations:** 1 Centre for Affective Disorders, Department of Psychological Medicine, Institute of Psychiatry, Psychology and Neuroscience, King's College London, London, UK; 2 South London and Maudsley NHS Foundation Trust, London, UK; 3 Departamento de Clínicas, Facultad de Medicina, Universidad Católica del Norte, Coquimbo, Chile; 4 Department of Neuroimaging, Institute of Psychiatry, Psychology and Neuroscience, King's College London, London, UK; 5 Cornell MRI Facility, Cornell University, Ithaca, New York, NY, USA

## Abstract

Major depression is associated with altered static functional connectivity in various brain networks, particularly the default mode network (DMN). Dynamic functional connectivity is a novel tool with little application in affective disorders to date, and holds the potential to unravel fluctuations in connectivity strength over time in major depression. We assessed stability of connectivity in major depression between the medial prefrontal cortex (mPFC) and posterior cingulate cortex (PCC), key nodes in the DMN that are implicated in ruminative cognitions. Functional connectivity stability between the mPFC and PCC over the course of a resting-state functional magnetic resonance imaging (fMRI) scan was compared between medication-free patients with major depression and healthy controls matched for age, sex and handedness. We tested replicability of the results in an independent sample using multi-echo resting-state fMRI. The primary sample included 20 patients and 19 controls, while the validation sample included 19 patients and 19 controls. Greater connectivity variability was detected in major depression between mPFC and PCC. This was demonstrated in both samples indicating that the results were reliable and were not influenced by the fMRI acquisition approach used. Our results demonstrate that alterations within the DMN in major depression go beyond changes in connectivity strength and extend to reduced connectivity stability within key DMN regions. Findings were robustly replicated across two independent samples. Further research is necessary to better understand the nature of these fluctuations in connectivity and their relationship to the aetiology of major depression.

## Introduction

Depression is a common illness with substantial negative consequences for sufferers and society.^1, 2^ A better insight into neurobiological changes contributing to symptom generation is a research priority to improve diagnosis and treatment.^3^

Neuroimaging has enhanced our understanding of the neurobiological mechanisms underlying depressive symptoms^3, 4, 5, 6^ by identifying potential alterations in the structural and functional brain networks.^4, 7^ The default mode network (DMN) is one system that has attracted great research interest in major depression. One reason for this relates to its importance in the generation of self-referential thoughts, negative rumination and depressive symptoms.^8^ A recent meta-analysis of static connectivity studies in major depression demonstrated hyperconnectivity within the DMN and between the DMN and fronto-parietal systems.^4^ Within the DMN, the subsystem connecting the medial prefrontal cortex (mPFC) with the posterior cingulate cortex (PCC) is considered pivotal in generating affective, self-directed judgements and thoughts.^8, 9^ Although heightened static functional connectivity has been shown in major depression within this subsystem in association with a ruminative cognitive style,^4, 10^ there is uncertainty about its functional temporal stability. Connectivity variability is a plausible mechanism in major depression to explain brain responses associated with cognitive demands and processing of emotions.^11^

There is, however, little dynamic functional connectivity research published to date^11, 12^ with only one study in major depression.^13^ This study showed increased variability in connectivity within the DMN between the mPFC and the insula, which correlated with a ruminative thinking style and coexisted with decreased variability between the mPFC and the parahippocampal gyrus.^13^ No effect was found between the mPFC and PCC in this study, despite these being crucial in the generation of self-directed negative affective cognitions, potentially due to the less powerful whole-brain analysis method used.

Hence, we specifically evaluated temporal connectivity variability between the mPFC and the PCC, a subsystem within the DMN, given its relevance to ruminative cognitions associated with depression.^8, 9^ Kaiser *et al.*^13^ observed increased connectivity variability between the mPFC and insula, part of the ‘salience network' that is known to influence synergistically this DMN subsystem when processing internally generated salient information,^14^ correlating with levels of rumination. Based on this finding that connections related to rumination show increased variability, we hypothesized increased connectivity variability in major depression between the mPFC and the PCC, correlating with ruminative cognitions. We tested this hypothesis first in a primary sample of medication-free participants with major depression selected to be free from psychiatric comorbidity. We then validated the robustness of this result by replicating the findings in an independent clinical sample. As a further test of the stability of the results in the presence of significant clinical heterogeneity, the validation sample included patients with major depression and comorbid anxiety disorders. Furthermore, the robustness of the neuroimaging results to non-neural artefacts was ensured by utilizing multi-echo functional magnetic resonance imaging (fMRI), a recent development that is superior to traditional de-noising methods.^15^

## Materials and methods

### Participants

The right-handed participants aged 18–65 were recruited from the local community using online advertisements^16^ and waiting lists of local psychological therapy services. Given the novelty of the method, we were unable to determine an expected effect size *a priori*. However, the sample size was chosen to be consistent with other studies in the area and we used a validation sample to ensure our results were robust. All patients met Diagnostic and Statistical Manual for Mental Disorders IV criteria for unipolar major depression (current or recurrent episode), as determined by clinical interview based on the Mini International Neuropsychiatric Interview.^17^ In the primary sample, comorbid conditions were excluded. For the validation sample, comorbid anxiety disorders were allowed alongside major depression. Depression severity was assessed with the Montgomery–Åsberg Depression Rating Scale^18^ and a score ⩾18 was required for inclusion. Raters for both samples were trained on an independent sample of patients and demonstrated high inter-rater reliability (Intraclass correlation coefficient=0.96, *P*=0.004). The severity of anxiety symptoms was assessed using the Hamilton Depression Rating Scale 17 anxiety/somatization factor items (anxiety subscale)^19, 20^ and handedness with the Edinburgh Handedness Inventory.^21^ Trait rumination was assessed using the Rumination Response Scale (RRS),^22^ a 22-item self-report measure. Patients were not receiving any form of treatment, psychological or pharmacological, at the time of scanning and were medication-free for ⩾2 weeks (⩾4 weeks for fluoxetine) before MRI scanning. No subjects had been receiving treatment with medication requiring a longer washout period. Healthy controls were assessed to exclude personal and familial (first-degree relatives) psychiatric history. Exclusion criteria for all the subjects included history of head injury, illicit substance use in the preceding two months, unstable medical illness, any treatment with potential psychotropic properties or interference with participants' safety or data interpretation, pregnancy or other contraindications for scanning.

### Ethics approval

The research was approved by the local ethics committee. The subjects provided written informed consent and were compensated financially for participating.

### fMRI acquisition

The data for each sample were acquired on two identical GE MR750 3-Tesla scanners with 12-channel radiofrequency head coils. The participants fixated on a cross with their eyes open for the scan duration. For the primary sample, a 6-min resting-state scan using a T2*-weighted echo-planar imaging sequence was acquired (repetition time=2000, echo time=30 ms, field of view=22.1 cm, flip angle=75°, 39 slices, resolution=3.3 mm^3^). The cardiac signals and respiratory information were also recorded. For the validation sample, the data were acquired using an 8-min multi-echo sequence (repetition time=2300 ms, echo time=12.7/31/48 ms, field of view=24 cm, flip angle=90°, 33 slices, resolution=3.75 × 3.75 × 4.2 mm). An identical high-resolution T1-weighted structural image was acquired for both the samples.

### fMRI preprocessing

The data were pre-processed with custom Nipype (http://nipy.org/nipype/) scripts, using tools from SPM12 (http://www.fil.ion.ucl.ac.uk/spm), FSL 5.0.9 (http://fsl.fmrib.ox.ac.uk/), AFNI (https://afni.nimh.nih.gov/afni/), along with custom code (available upon request). The first four volumes of the functional series were discarded to allow for equilibration effects. Slice timing correction was performed and the images were realigned and co-registered to the structural image using the normalized mutual information method in SPM12. For the primary sample, physiological signals (cardiac and respiratory) were regressed from the data using AFNI's RETROICOR^23^ tool. For the validation sample, multi-echo data were pre-processed using the multi-echo independent component analysis tool in AFNI^15^ to isolate components in the signal representing true blood oxygen level dependent (BOLD) signal. This was used in place of RETROICOR as it has been shown to be a more effective method of de-noising.^15^ The remaining processing steps were identical for both samples for consistency. Six motion parameters (three translation, three rotation, determined from the middle echo image for the validation sample) plus time series extracted from the white matter and cerebrospinal fluid regions were regressed out of the data. Data were then temporally filtered from 0.008 to 0.09 Hz before being demeaned, de-trended and smoothed with a 6 mm full width at half maximum kernel. Thus, preprocessing for both samples was identical except for the method of de-noising used.

### Motion scrubbing

As even minimal head motion can affect correlations calculated from resting-state data when not controlled for,^24^ time points exhibiting excessive motion were scrubbed from the BOLD time series.^24^ Motion at each time point was assessed using root mean square (RMS) intensity difference between volumes (REFRMS) and DVARS^24^ as calculated using the FSL motion outliers tool with default thresholds. As directly removing time points would affect the length of the sliding window, and hence dynamic connectivity estimates, we instead interpolated time points showing substantial motion using third-order b-spline interpolation. To compare motion estimates between samples, we used both total distance travelled and framewise displacement.^24^ All the analyses were performed on the scrubbed, pre-processed data.

### Region of interest definition

We performed group canonical independent component analysis,^25^ implemented in Nilearn and using 20 clusters, to identify the DMN components. The clusters centred on the posterior cingulate and mPFC regions in the DMN component were used to create regions of interest (ROIs) for the connectivity variability analysis. A 10 mm diameter sphere was created based on the peak of each cluster in the independent component analysis, and mean time series were extracted from each of these ROIs. This procedure was performed independently for the two samples to identify sample-specific ROIs. These ROIs were used in place of the entire cluster to provide a more consistent signal and avoid contamination from surrounding areas. To ensure that results were specific to these regions rather than being a global pattern, or caused by non-neural factors, we performed a negative control analysis between these regions and a 10 mm spherical ROI in the medial primary motor cortex (Montreal Neurological Institute coordinates=−1, −8, 63), a region not previously linked to depression.

### Sliding window correlation analysis

The sliding window analysis was performed using custom Python (https://www.python.org/) scripts. The data were split into 40 s Gaussian moving windows, staggered by one repetition time, created using a Gaussian kernel with a standard deviation of 8 s (see Supplementary Methods for a detailed discussion of the sliding window methodology). This time period has been shown to be appropriate for characterizing dynamic functional connectivity^26^ and provides a fine-grained picture of temporal changes in connectivity. For each window, correlations were computed between variance-normalized time series from the two regions using Pearson correlations, the results of which were then transformed to *Z*-scores. The variability of these correlations was calculated as their standard deviation, and subjects with outlying correlation variability values (±3 standard deviations from the mean) were removed. We also calculated the static functional connectivity strength between these regions using the entire, non-windowed time series to understand the relationship between static and dynamic functional connectivity. Further statistical analyses were performed using R.^27^ All the group comparisons and correlations were adjusted for age, sex and head motion (total distance travelled), and were corrected for the number of comparisons (mPFC–PCC and two negative controls) using false discovery rate correction.

We assessed relationships between functional connectivity variability and clinical measures including depression and anxiety severity scores, time since illness onset and RRS in Pearson partial correlations.

### Voxel-based morphometry

We also compared grey matter volumes in the chosen ROIs between patients and controls using voxel-based morphometry to examine the co-existence of volumetric changes that may explain changes in connectivity. High-resolution T1-weighted structural images (repetition time=7.31 ms, time to echo=3.02 ms, 256 × 256 matrix, 196 slices, voxel size=1.2 × 1.05 × 1.05 mm, for both the samples) were pre-processed using voxel-based morphometry in SPM12 (www.fil.ion.ucl.ac.uk/spm) for both the samples. Images were segmented into different tissue types and processed with DARTEL^28^ before being normalized to Montreal Neurological Institute space. The modulated grey matter images were then smoothed with an 8 mm full width at half maximum Gaussian kernel. Grey matter volume was next compared between groups within the same mPFC and PCC regions of interest used in the functional connectivity analysis with a two-sample *t*-test. A cluster-defining voxelwise threshold of *P*<0.001 uncorrected was used, with a clusterwise threshold of 0.05 false discovery rate corrected. Total grey matter volume was also calculated based on the segmented maps created in SPM (thresholded at grey matter volume probability >0.5) and compared between the groups.

## Results

### Participants

**Figure 1:**
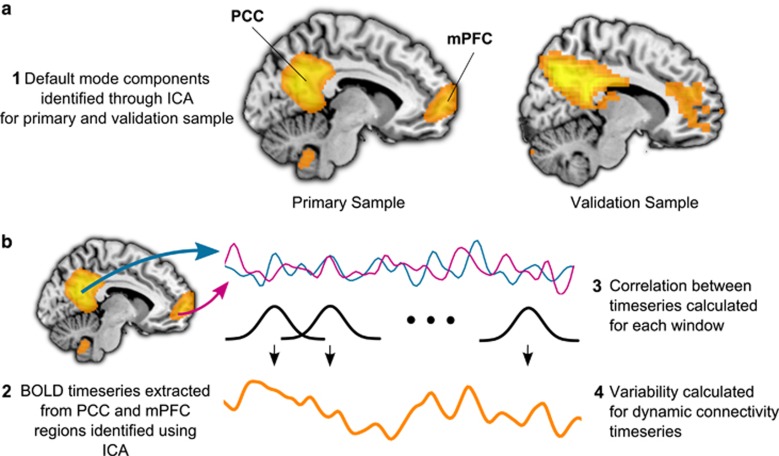
Illustration of the dynamic functional connectivity analysis method. In the primary sample, sample-specific Montreal Neurological Institute (MNI) coordinates for the posterior cingulate cortex (PCC) were 2, −62, 22 and for the medial prefrontal cortex (mPFC) 4, 60, 0. In the validation sample, MNI coordinates were 6, −44, 11 and 2, 60, −4, respectively. (**a**) Default mode network (DMN) components for each sample identified using group independent component analysis (ICA), showing clusters in the mPFC and PCC. (**b**) Illustration of the dynamic functional connectivity method. BOLD, blood oxygen level dependent.

Twenty patients with unipolar major depression in the primary study and 19 in the validation study were sex and age matched with 19 healthy controls in the primary sample and 20 in the validation sample (Table 1). Three and nine subjects were recruited through psychological therapy services in the primary and validation samples, respectively, while the remaining participants were recruited from the community. A healthy participant from the validation sample was removed from the analyses due to outlying connectivity variability values. Figure 1 shows coordinates for the selected ROIs.

### Connectivity variability in major depression

**Figure 2:**
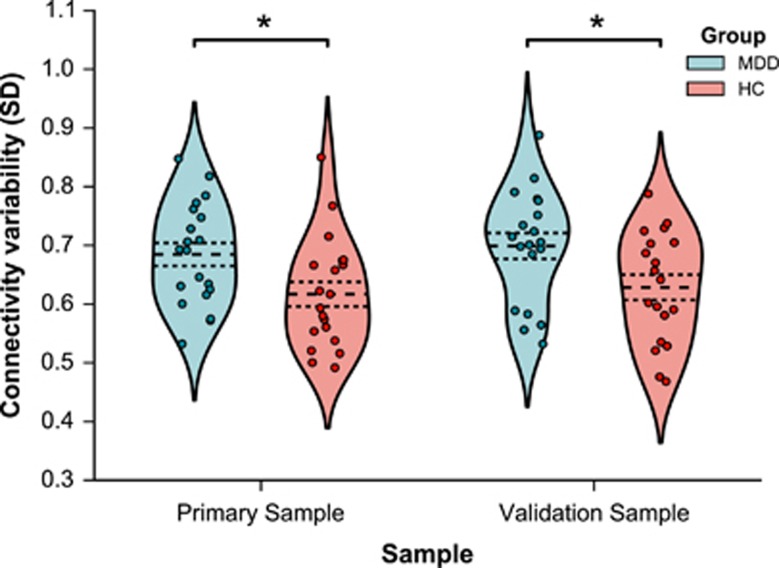
Variability in connectivity strength for patient and healthy control groups in both samples. Plots represent the distribution of data for each group, along with individual data points. Dashed lines represent means and standard errors. *P<0.05 (*P*=0.044 for the primary sample and *P*=0.048 for the validation sample). HC, healthy controls; MDD, major depressive disorder.

Connectivity variability, expressed as the standard deviation of connectivity strength between the mPFC and the PCC (Figure 2), was significantly greater in patients with major depression versus healthy controls (*t*(37)=2.56, *P*=0.044, *d*=0.82). This effect was successfully replicated in the validation sample (*t*(36)=2.53, *P*=0.045, *d*=0.82) supporting the coherence of the model in identifying consistently greater connectivity variability across samples irrespective of clinical heterogeneity.

There were no group differences in connectivity variability in either sample between the mPFC and primary motor cortex, chosen as a negative control region (primary sample: *t*(37)=0.79, *P*=0.44, validation sample: *t*(36)=1.85, *P*=0.22), or between the PCC and primary motor cortex (primary sample: *t*(37)=1.76, *P*=0.17, validation sample: PCC: *t*(36)=0.63, *P*=0.99), suggesting that results did not reflect global instability. No differences in static connectivity strength between mPFC and PCC were found between patients and controls in either sample (primary sample: *t*(37)=0.33, *P*=0.74, *d*=0.11, validation sample: *t*(36)=0.73, *P*=0.47, *d*=0.24).

### Connectivity variability and cognitive style

**Figure 3:**
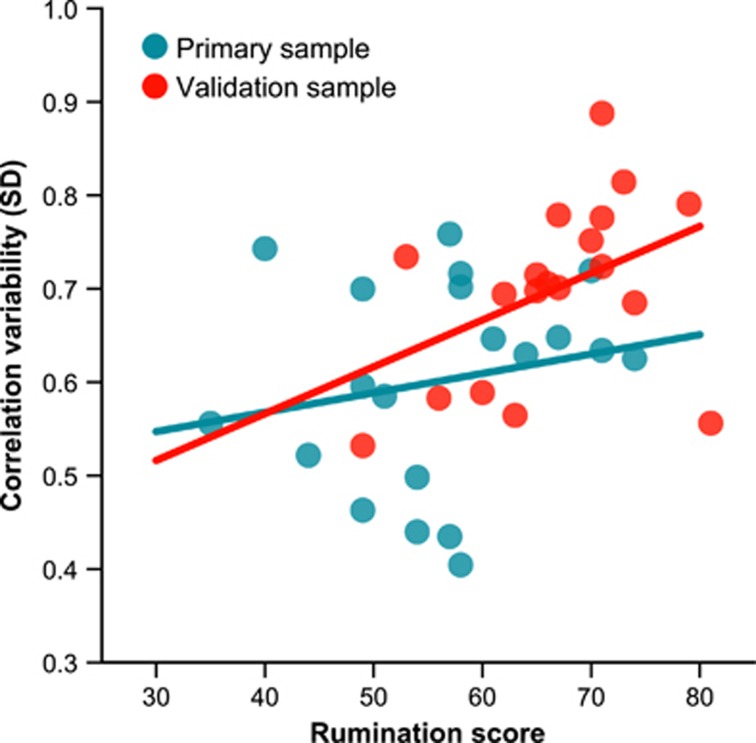
Correlation between connectivity variability and ruminative response style (RRS) score in major depression in the primary and validation sample. RRS, Rumination Response Scale.

A positive correlation of RRS with connectivity variability was noted in the validation sample (*r*(14)=0.51, *P*=0.045, Figure 3), and not the primary sample (*r*(15)=0.18, *P*=0.48). The relationship did not remain significant in a pooled analysis with both groups combined and sample as a covariate (*r*(34)=0.30, *P*=0.075). We did not find significant correlations between RRS scores and static connectivity strength in either sample (primary sample: *r*(15)=0.31, *P*=0.22, validation sample: *r*(14)=−0.13, *P*=0.62).

### Correlations with clinical variables

Correlations between connectivity variability and depressive symptom severity were not significant (primary sample: *r*(15)=0.37, *P*=0.14, validation sample: *r*(14)=0.23, *P*=0.40). The same pattern of results was observed between correlation variability and anxiety symptoms scores, as measured by the anxiety subscale of the Hamilton Depression Rating Scale (sample A: *r*(15)=0.11, *P*=0.66, sample B: *r*(14)=-0.23, *P*=0.39). The time since illness onset was not significantly correlated with connectivity variability in either sample (sample A: *r*(15)=0.25, *P*=0.33, sample B: *r*(14)=0.16, *P*=0.55).

### Grey matter volumes

There were no differences between groups in grey matter volume in the chosen regions of interest (no significant clusters at *P*<0.001, false discovery rate corrected). There were also no differences in the total grey matter volume between groups (primary sample: *t*(37)=1.43, *P*=0.16, validation sample: *t*(36)=0.79, *P*=0.43).

### Head motion

There were no significant differences in the total distance travelled between the patients and controls in the primary sample (*t*(37)=−1.31, *P*=0.20) or the validation sample (*t*(36)=0.56, *P*=0.58). When looking at framewise motion measures, there were no significant differences between the groups in mean framewise displacement (primary sample: *t*(37)=0.88, *P*=0.38, validation sample: *t*(36)=0.26, *P*=0.80) or maximum displacement (primary sample: *t*(37)=0.42, *P*=0.68, validation sample: *t*(36)=1.48, *P*=0.15). There was also no significant difference between groups in the number of interpolated time points (primary sample: *U*(37)=173, *P*=0.64, non-parametric test used due to non-normally distributed data, validation sample: *t*(36)=1.60, *P*=0.12), or in the thresholds used for detecting outlying time points for either the primary sample (REFRMS: *t*(37)=−0.14, *P*=0.89, DVARS: *t*(37)=0.42, *P*=0.68) or the validation sample (REFRMS: *t*(36)=0.67, *P*=0.51, DVARS: *t*(36)=0.71, *P*=0.48).

There were also no significant correlations in the validation sample between rumination scores and total distance travelled (*R*(17)=0.13, *P*=0.59), mean displacement (*R*(17)=-0.09, *P*=0.70), maximum displacement (*R*(17)=0.17, *P*=0.48), or the number of interpolated time points (*R*(17)=0.11, *P*=0.66), indicating that the observed relationship between connectivity variability cannot be explained by motion. There were no significant relationships between thresholds used to detect outlying time points and rumination scores (REFRMS *R*(17)=0.11, *P*=0.65, DVARS: *R*(17)=0.35, *P*=0.15).

## Discussion

We compared variability in connectivity strength within the DMN between medication-free individuals with major depression and matched healthy controls. We found that connectivity between the mPFC and PCC, two key nodes in the DMN, was significantly more variable in currently symptomatic patients with major depression. Furthermore, the validity of the results was confirmed in an independent sample of individuals with significantly more clinical heterogeneity, and using multi-echo acquisition parameters to limit the impact of non-neural signals. We believe this is the first time that greater connectivity variability has been reported in major depression in this DMN subsystem, complementing findings of reported abnormal dynamic connectivity in other brain regions in major depression^13^ and other psychiatric conditions.^11, 29, 30^

In agreement with Kaiser *et al.*,^13^ we demonstrated that the increased variability in neural connectivity originating from the mPFC correlates with a ruminative thinking pattern, indicating a possible synergy of this DMN subsystem with the ‘salience network' represented by the insula.^14^ This previous study, a whole-brain analysis, demonstrated altered connectivity variability between the mPFC and other DMN regions, but not specifically the PCC,^13^ and variability in connectivity between the mPFC and parahippocampal gyrus was reduced. This might be explained by the more limited power in whole-brain analyses due to the necessary correction for multiple comparisons. Another possible explanation is that depression is associated with increased connectivity variability in the central DMN (including the PCC), while variability in the ventral system (including the parahippocampal gyrus) is reduced.

At present, the biological significance of time varying properties of connectivity is not well understood.^12^ One potential explanation for the observed greater connectivity variability is that it results from reduced structural connectivity in the DMN as suggested by previous research linking structural integrity with connectivity variability.^31^ However, our recent meta-analysis reported that structural integrity is maintained in the tracts connecting these regions in major depression,^7^ suggesting that such an explanation is unlikely, further supported by our finding of no alteration in grey matter volume in the ROIs in these patient samples. It is also possible that greater connectivity variability reflects primary alterations in neuronal communication rather than occurring secondary to aberrant structural connectivity, a suggestion that is in line with preclinical work showing primary abnormalities of neural processing in circuits relevant to depression.^32^ Clarifying the precise meaning of connectivity variability, and how it relates to connectivity strength, at a neural level will be an important task for future research.

We also tested the hypothesis that a ruminative cognitive style, linked with depression,^8, 33^ potentially explains the observed abnormalities in connectivity variability between mPFC and PCC. We observed a direct positive correlation between levels of intrusive, self-generated, ruminative thoughts and variability in connectivity within this network. This was, however, present only in the validation sample. This discrepancy in the results can be explained by issues related to statistical power combined with variability in the samples clinical characteristics. Patients in the validation sample were in fact characterized by higher rumination scores and comorbid anxiety disorders. It is possible that this result is reflective of a stronger link between anxiety symptoms and rumination,^34^ and further work could clarify this. The difference between samples may also be related to differences in image acquisition and preprocessing. It is also possible that connectivity variability might be more closely linked to ‘state' rumination levels occurring at the time of scanning rather than more ingrained ‘trait' measures such as the RRS or to state anxiety, which we did not assess here. This is supported by work adopting post-scan cognitive style reports in healthy volunteers.^35^ This study suggested a positive correlation between variability in the DMN and reported daydreaming (a related phenomenon to rumination) during MRI scanning, consistent with our findings.

We found no association in either sample between variability and depression severity scores. This may suggest that increased variability is not directly related to depressive symptoms. However, it is possible that this could be due to the Montgomery–Åsberg Depression Rating Scale, our measure of symptom severity, being weighted towards somatic rather than cognitive symptoms of depression. Previous research suggests that alterations in DMN connectivity are more likely to be related to psychological symptoms such as negative self-related cognitions.^33^ Another possible explanation is that the relationship between biological disease mechanisms and symptoms is complex, and tend not to correlate linearly with one another.^11^

We found no alterations in static connectivity strength in both our samples in agreement with some studies^36^ but not others.^37^ Discrepancies in the findings can be explained by methodological differences in connectivity measurements, the proneness of neuroimaging data to type I/II error based on their relatively moderate sample sizes as indicated by recent meta-analyses^4^ and multiple sources of heterogeneity intrinsic to major depressive disorders. We adopted a cross-validation method applied for the first time to this type of data to help enhance robustness of the findings. Based on our experience, future studies could consider a similar approach with larger samples, perhaps in the context of collaborative mega-analyses^38^ to reduce statistical bias and increase power.

In addition to this potential link between connectivity instability and alterations in cognitive or emotional state, such as rumination, instability may also reflect increased noise or alterations in neural dynamics. Simulation studies have indicated that patterns of synchronization and desynchronization in neuronal populations lead to fluctuations in functional connectivity as measured using fMRI,^39^ while noise-driven neuronal simulations produce switches between states of functional connectivity.^40^ Notably, simultaneous electroencephalography and fMRI have shown that changes in the BOLD functional connectivity mirror electroencephalography power fluctuations^41^ further indicating that variations in the functional connectivity are reflective of neuronal processes. It is possible that fluctuations in connectivity may reflect underlying changes in neural synchrony, a key process in inter-regional communication, which has been proposed to be affected in a range of psychiatric disorders.^42^

It has previously been suggested that hyperconnectivity implies lower variability in connectivity.^13, 43^ However, this is at odds with our results as we did not find group differences in static connectivity. Hence, the relationship between dynamic and static connectivity appears complex, with dynamic functional connectivity providing distinct information about network communication in a state of pathology independent from and beyond that of static connectivity. This echoes findings from previous research that attempted to classify patients with schizophrenia, bipolar disorder and healthy controls based on functional connectivity, indicating that classification using a combination of static and dynamic connectivity tends to be more accurate than static connectivity alone.^11, 44^

A notable strength of this study is the control of non-neural influences on the data. The resting-state fMRI analyses are susceptible to influence from confounding factors such as motion and physiological variables,^24^ and this is especially pertinent here in view of the possibility that physiological characteristics such as heart rate may differ between groups due to increased anxiety in patient groups. Consequently, we rigorously controlled for these in a number of ways, including correction for cardiac and respiratory signals, and motion scrubbing.^24^ In our replication sample we used multi-echo fMRI with independent component analysis-based de-noising, which is more effective than traditional de-noising methods,^15^ providing further evidence that our original results were not a result of non-neural influences. Moreover, our negative control analyses indicate that our findings were specific to the disease-relevant network under investigation, and groups did not differ on motion parameters.

Further strengths of the present study are the inclusion of medication-free patients, suggesting that the findings are not due to pharmacological effects. In addition, many patients in the primary sample were medication-naive and had experienced few, if any, past depressive episodes, making it less likely that the effects observed are cumulative effects of illness or previous treatment. The demonstration of greater connectivity variability in the validation sample, which included patients with more chronic and heterogeneous illness and used multi-echo fMRI, increases confidence in the reliability of the findings, which are unlikely to be attributable to idiosyncrasies of a specific sample or methodological artefacts. This is particularly important in view of concerns regarding poor reproducibility in research.^45^ Replication is an important step towards minimizing false associations and enhancing reliability of results,^45^ and the reproducibility demonstrated here suggests that alterations in dynamic functional connectivity are robust. Future replications of these results are warranted, and it would be of particular interest to examine connectivity variability in non-symptomatic individuals with depression and ‘at risk' samples to test whether it is a feature of the depressive state, a marker of vulnerability to depression or a ‘scar' effect.

One limitation of this study is the focus on two isolated regions of the DMN. We chose these regions given their key role in the DMN, and association with rumination in major depression.^8, 9^ Focusing on *a priori* regions of interest nevertheless increased the power to detect changes given the relatively small sample sizes. Additional analyses to confirm the replicability of previous findings, such as altered dynamic connectivity with the insula, would have been of interest; however, we chose to focus on one particular component of the DMN to reduce the likelihood of type II errors due correction for multiple comparisons with a small sample.

Furthermore, alternative methods to sliding window analyses, such as coherence-based methods, have been proposed that may provide more accurate estimations of dynamic functional connectivity.^12^ Given their novelty, we have however chosen to use a more established method that has been evaluated in numerous studies. It is also possible that using different window lengths may affect the analysis. We believe the 40 s windows used here is an optimal length for detecting alterations in dynamic functional connectivity (see Supplementary Material for further discussion of this issue). In addition, our imaging sequences were only 6 and 8 min long for the primary and replication sample respectively. This may have limited our ability to detect less-frequent fluctuations in connectivity. Furthermore, our replication data and analysis differed slightly from our primary sample in acquisition and de-noising methods, making this more of a conceptual than methodological replication. Nevertheless, the fact that our results were largely consistent despite these dissimilarities suggests that the effect is robust.

In view of the presence of a range of anxiety disorders in the validation sample, it is not possible to determine whether altered connectivity variability might be a common abnormality present in both major depression and anxiety disorders rather than being specifically associated with depression. High comorbidity rates between anxiety and depressive disorders and the co-occurrence of anxiety symptoms in major depression are frequent findings in clinical practice.^1^ This makes the differentiation challenging to establish. Lastly, it should be noted that the sample sizes used here were relatively small. Nonetheless, the consistency of our results across two independent samples indicates that our results are unlikely to be spurious.

In conclusion, our study indicates that major depression is associated with reduced stability of connectivity within the DMN in key regions relevant to the generation of ruminative cognitions. This could represent an intrinsic neural property of this illness and a potential network-specific brain abnormality not previously explained by structural abnormalities or static functional connectivity. The results were replicated in a second independent sample, indicating that they are robust. Ruminative cognitive style might partially explain the results in keeping with cognitive models of depressive disorders. This work adds to functional connectivity research in affective disorders by validating new findings across different samples with novel fMRI analysis techniques. Further work investigating trait markers of vulnerability to depression and connectivity variability in remitted patients are necessary to establish the specificity of these findings to the depressed state. Similarly, longitudinal studies are required to explore the effect of treatment on these abnormalities.

## Figures and Tables

**Table 1:** Samples clinical characteristics

	*Major depression*	*Healthy control*	P
*Primary sample*			
*n*	20	19	
Age, years	29.55 (6.59)	30.05 (6.71)	0.81
Male/female	2, 18	2, 18	1
MÅDRS	27.25 (4.24)	0.95 (1.39)	<0.001
HDRS anxiety subscale	4.95 (2.48)	0.21 (0.42)	<0.001
RRS	56.00 (10.23)	29.67 (6.44)	<0.001
Time since illness onset, years	6.35 (6.41)	—	—
Comorbid diagnoses	None	—	—
Hospitalizations	0	—	—
Number of episodes	1.5 (1.25)	—	—
			
*Validation sample*			
*n*	19	19	
Age, years	32.34 (10.62)	31.91 (10.30)	0.90
Male/female	7, 12	6, 13^a^	0.73
MÅDRS	30.74 (7.31)	1.37 (1.86)	<0.001
HDRS anxiety subscale	7.16 (1.30)	0.31 (0.58)	<0.001
RRS	66.47 (8.22)	30.63 (6.83)	<0.001
Time since illness onset, years	13.50 (8.26)	—	—
Comorbid diagnoses	*N*=12 (9 GAD, 5 SAD, 4 OCD, 2 PD, 2 PTSD, 1 historic substance abuse)	—	—
Hospitalizations	4	—	—
Number of episodes	4 (2.5)	—	—

Abbreviations: GAD, generalized anxiety disorder; HDRS, Hamilton Depression Rating Scale (17 item); MÅDRS, Montgomery-Åsberg Depression Rating Scale; OCD, obsessive compulsive disorder; PD, panic disorder; PTSD, post-traumatic stress disorder; RRS, Ruminative Response Scale; SAD, social anxiety disorder.

a Demographics for the validation sample represent the 19 healthy controls included in the final analysis. The samples did not differ on depression severity (*t*(37)=−1.83, *P*=0.07). The validation sample had a significantly higher anxiety score (*t*(37)=3.45, *P*=0.001), time since illness onset (*t*(37)=2.62, *P*=0.01) and RRS score (*t*(37)=3.51, *P*=0.001) than the primary sample.

Values are reported as mean (standard deviations) for all variables except number of episodes where due to skewed data we report median (interquartile range).
