# Supplementary material for: Instability of default mode network connectivity in major depression: a two-sample confirmation study
Source: Transl Psychiatry. 2017 Apr 25;7(4):e1105–. doi: 10.1038/tp.2017.40 (PMC5416685; doi:10.1038/tp.2017.40)
Supplement: Supplementary Information [file tp201740x1.docx]

# Choice of window length for dynamic functional connectivity analyses

Here we outline the justification for the sliding window length and high-pass cut-off frequency used in our analyses. Given the novelty of this field, there remains considerable debate in the literature regarding optimal methods for assessing dynamic functional connectivity. We use simulated data to validate our analyses methods, and ensure we are measuring real and not spurious fluctuations in connectivity.

A particularly contentious issue is the choice of sliding window length, and its relationship to the frequency used when high-pass filtering the data. Recently, it has been suggested that dynamic functional connectivity analyses using short window lengths can lead to inaccurate estimates of correlation between timeseries (Leonardi & Van De Ville, 2015b). The authors of this paper initially stated that the combination of low high-pass cut-off frequencies and short window lengths can lead to positively correlated timeseries being estimated as negatively correlated in extreme cases; however, this has since been corrected to indicate that in this situation correlation coefficients will on average be underestimated to a small degree (Leonardi & Van De Ville, 2015a). Notably, this only occurs when one timeseries is phase lagged relative to the other. When there is no such phase lag, windowed correlation analyses are able to estimate correlation coefficients accurately.

The recommendations made by Leonardi & Van De Ville (2015b) are to either use a longer window (a minimum length in seconds of 1/f_mn_, where f_min_ is the minimum frequency of interest) or to high-pass the data at a higher frequency (e.g. 1/w, where w represents window length). However, both of these create additional problems in measuring dynamic functional connectivity. Firstly, longer windows will be unable to detect rapid changes in connectivity strength, both underestimating strong correlations and overestimating weak ones, while short windows are sensitive to such changes. We demonstrate this empirically using simulated data (Figure S1), across three window lengths: 40 seconds (as used here), 80 seconds (double the length used in this study), and 126 seconds (1/f_min_ with a high-pass filter of 0.008hz as used in this study). Notably, using a window length of 126 seconds reduces the variation in correlation coefficients at higher frequency connectivity oscillations to near-zero (Figure S1).


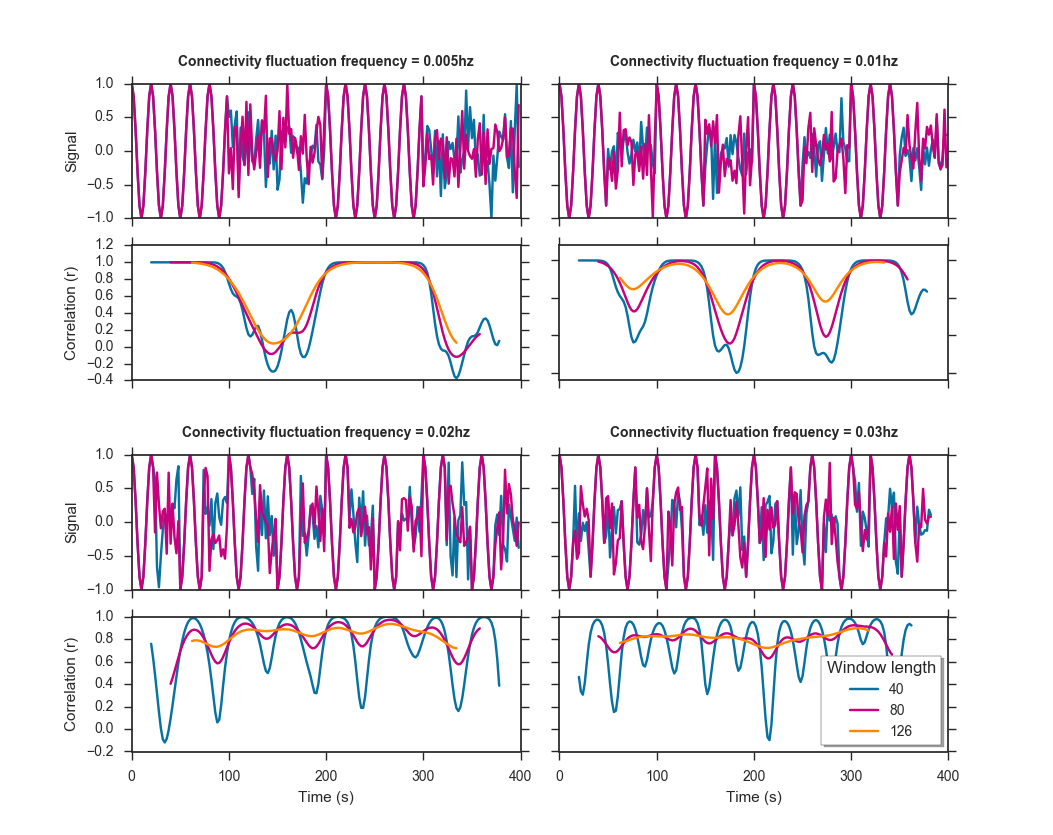


Figure S1. Simulated fluctuations in connectivity. Two timeseries are simulated that alternate between perfect correlation (two sinusoidal signals at 0.05hz) and no correlation (two white noise signals) at frequencies of 0.005hz, 0.01hz, 0.02hz, and 0.03hz, frequencies at which connectivity typically fluctuates (Allen et al., 2014). Correlations between timeseries are shown for three Gaussian window lengths: 40 seconds (as used in this study), 80 seconds, and 126 seconds (the approximate 1/f_min_ window length for a high-pass filter at 0.008hz, as used here).

Secondly, using a higher cut-off frequency when filtering the data will remove low frequency signals, which are important components of resting state networks. For example, with a window length of 40 seconds, as used here, the appropriate 1/w cut-off frequency would be 0.025hz. As can be seen from figure S2, the frequency distribution peaks at around this frequency; signals below this are prominent in the BOLD signal and would be lost if we used a lower cut off.

In their analyses, Leonardi & Van De Ville (2015a) demonstrate that when using a 40 second window (as chosen here), correlations using sliding windows will on average be underestimated when there is significant phase lag between timeseries. Their analysis was based on boxcar windows, however we chose to use Gaussian windows here. As shown in Figure S3, when using Gaussian windows, the decay in correlation coefficients with shorter window lengths is less smooth, but at the window length used here is only 93.8% of the true correlation magnitude at the lowest frequency included in this study (0.008hz). Even when using 40 second boxcar windows, as in the study by Leonardi & Van De Ville (2015b), the estimated correlation is still 86.1% of the true correlation magnitude. As such, the effect of these short windows on correlation estimates is minimal, and we are confident that our choice of window length will not lead to spurious estimates of connectivity.


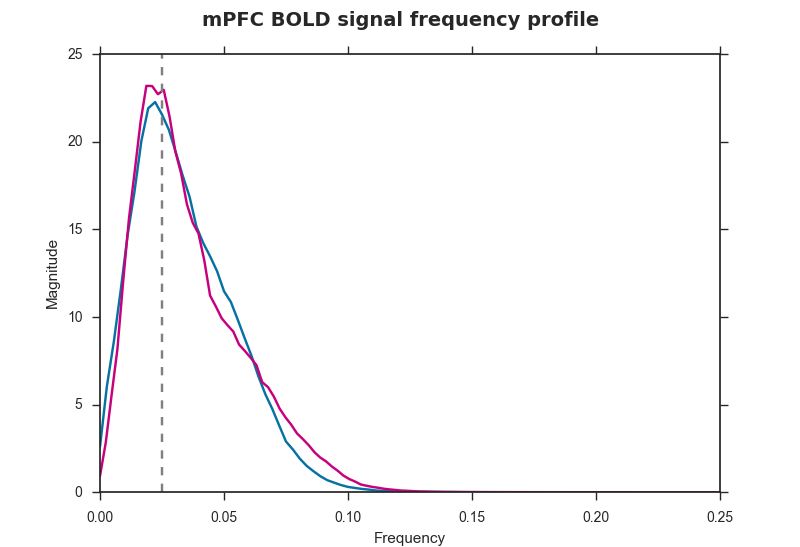


Figure S2. Magnitude as a function of frequency for the mPFC ROI, after bandpass filtering between 0.008hz and 0.09hz, averaged across all subjects. The 1/w frequency for 40 second windows is shown at 0.025hz.

As mentioned previously, the problems identified by Leonardi & Van De Ville (2015b) only become apparent when there is substantial phase lag between the timeseries used to calculate connectivity in the low frequency domain (i.e. below 1/w). To ensure that this could not explain our results, we compared mPFC-PCC phase lags between groups using timeseries band-passed between the lowest frequency used in our original filtering (0.008hz) and the 1/w frequency (0.025). Phase lag was measured using the phase-locking value (PLV, Lachaux, Rodriguez, Martinerie, & Varela, 1999), an index of the average difference in phase between two signals. There were no differences in PLV between patients and controls in either the original sample (*t*(37) = 0.79, *p* = 0.44), or the replication sample (*t*(36) = 1.38, *p* = 0.18), indicating no difference in low frequency phase between groups that could explain our results.


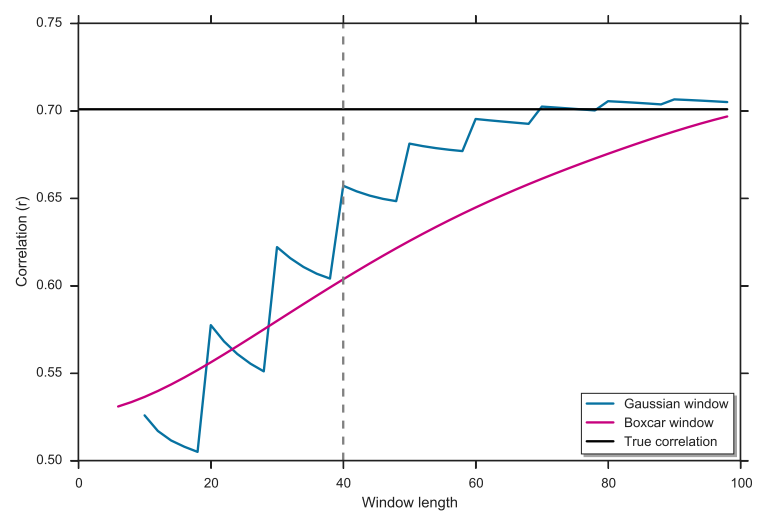


Figure S3. Correlation strength as a function of window length for a pair of sinusoidal waves at 0.008hz with a phase lag of π/4, with the window length used in this study (40 seconds) indicated.

In view of these advantages and disadvantages, we feel that the sensitivity to relatively high-frequency changes in connectivity strength and the ability to include low-frequency BOLD signals outweigh the problems with underestimation of correlation coefficients associated with short window lengths. As such, we believe that the 40 second windows here provide an effective method for measuring dynamic functional connectivity.

As a final point, it is important to note that these methods may not be applicable to all studies of dynamic functional connectivity. For example, studies of task-modulated connectivity may focus on high-frequency changes in connectivity and as such there would be fewer problems associated with using higher cut-off frequencies when high-passing the BOLD data.

## Supplemental references

Allen, E. A., Damaraju, E., Plis, S. M., Erhardt, E. B., Eichele, T., & Calhoun, V. D. (2014). Tracking Whole-Brain Connectivity Dynamics in the Resting State. *Cerebral Cortex*, *24*(3), 663–676. http://doi.org/10.1093/cercor/bhs352

Lachaux, J. P., Rodriguez, E., Martinerie, J., & Varela, F. J. (1999). Measuring phase synchrony in brain signals. *Human Brain Mapping*, *8*(4), 194–208.

Leonardi, N., & Van De Ville, D. (2015a). Erratum to ‘On spurious and real fluctuations of dynamic functional connectivity during rest’. *NeuroImage*, *104*, 464–465. http://doi.org/10.1016/j.neuroimage.2014.10.045

Leonardi, N., & Van De Ville, D. (2015b). On spurious and real fluctuations of dynamic functional connectivity during rest. *NeuroImage*, *104*, 430–436. http://doi.org/10.1016/j.neuroimage.2014.09.007
